# Late Onset of Entrectinib-Related ST Elevation in the Setting of Alcohol Intoxication in a Patient With Metastatic Non-small Cell Lung Cancer: A Case Report and Literature Review

**DOI:** 10.7759/cureus.98345

**Published:** 2025-12-02

**Authors:** Chenfan Xia, Suzanne Kosmider

**Affiliations:** 1 Department of Oncology, Western Health, Melbourne, AUS

**Keywords:** alcohol-related disorder, cancer treatment side effects, cardiac arrhythmia, entrectinib, non-small cell lung carcinoma (nsclc), ros1 gene fusion, ros1-positive nsclc, st elevation ecg, targeted therapy, tyrosine kinase inhibitor (tki)

## Abstract

This report describes a case of a 56-year-old male with non-small cell lung cancer who had been on entrectinib 600 mg daily for five years. He presented with an acute onset of chest pain associated with transient ST elevation and troponin rise in the context of alcohol intoxication. He was investigated with a coronary artery angiogram, a transthoracic echocardiogram, and a cardiac magnetic resonance imaging, which were all unremarkable. The ST elevation resolved after withholding entrectinib for two days. Given that no other cause was identified, it suggests that entrectinib may be the potential cause. Considering the patient's good response to entrectinib in the past, we recommenced entrectinib at a reduced dose of 400 mg daily after withholding it for three weeks. This late onset of ECG changes after such a prolonged treatment duration has not been reported previously, and the underlying mechanism remains unclear. It is also uncertain whether heavy alcohol intake is a contributing factor. Further research is needed to investigate the long-term cardiac toxicity of entrectinib and the potential interactions between entrectinib and alcohol.

## Introduction

ROS proto-oncogene 1 (ROS1 gene) fusions are present in various tumour types, including 1% to 2% of non-small cell lung cancer (NSCLC) [1]. The resulting oncoprotein causes continuous kinase activation and downstream signalling, and leads to tumour growth [2]. It is more common in younger individuals, females, and non-smokers. Entrectinib is a tyrosine kinase inhibitor that has been used for treating ROS1 fusion-positive NSCLC, demonstrating significant systemic and intracranial efficacy, along with prolonged survival benefit [1]. However, there have also been reports of serious treatment-related adverse events. For example, 2% of patients developed cardiac disorders, including QT prolongation, myocarditis, and cardiac failure [2]. There are also case reports of entrectinib-associated ST elevation. The exact mechanism is not yet fully understood, but it may be due to dysfunction of iron channels [3].

This report describes a case of a 56-year-old male with NSCLC who had been on entrectinib for five years. He presented with an acute onset of chest pain associated with transient ST elevation and troponin rise in the context of alcohol intoxication. He was investigated with a coronary artery angiogram, a transthoracic echocardiogram, and a cardiac magnetic resonance imaging (MRI), which were all unremarkable. The ST elevation resolved after withholding entrectinib for two days, and no other cause was identified, suggesting that entrectinib may be the potential cause. It is also unclear why this event occurred after five years of treatment and whether alcohol consumption was a contributing factor. This late onset of ST elevation after such a prolonged treatment duration has not been reported previously. Further research is needed to investigate the long-term cardiac toxicity of entrectinib and the potential interactions between entrectinib and alcohol.

## Case presentation

**Figure 1:**
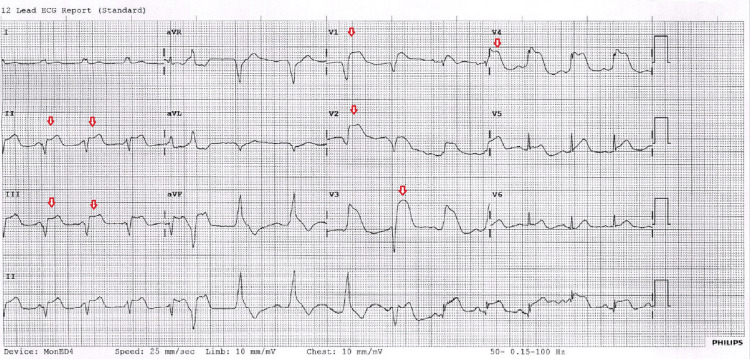
Electrocardiogram (ECG) showed significant ST elevation in both the anterior and inferior leads, with wide QRS complexes (red arrows).

A 56-year-old male presented with an acute onset of central chest pain for three hours and three episodes of witnessed collapsing in the setting of heavy alcohol intake. His electrocardiogram showed significant new ST elevation in both the anterior and inferior leads, with new wide QRS complexes, suggesting an ST-elevation myocardial infarction (Figure 1).

He was treated with aspirin and heparin while en route by ambulance and was subsequently taken to the emergency department.

The patient had a medical history of metastatic lung adenocarcinoma, which was diagnosed seven years ago. He had been on a third-line treatment of entrectinib 600 mg daily for the past five years with a complete radiological response. Additionally, he had a history of depression, for which he was taking mirtazapine. The patient also had intermittent heavy alcohol consumption for the past 16 years, and prior to this presentation, he was consuming 16 standard drinks per day for five consecutive days due to social stress.

Upon arrival at the emergency department, the patient's blood pressure was 150/137 mmHg, heart rate was 95/minute, respiratory rate was 20/minute, and oxygen saturation was 97% on room air. He was intoxicated, with a breathalyzer reading of 0.117; however, he was still responding appropriately with a Glasgow Coma Scale score of 15. Plasma high-sensitive troponin I levels peaked at 506 ng/L (normal value <21 ng/L) (Table 1).

**Table 1:** High-sensitivity troponin I results. Troponin peaked on presentation.

Time of sampling	Result (ng/L)	Reference range (ng/L)
On presentation	506	<21
Next day	226	<21

He underwent an emergency coronary artery angiogram, which showed normal coronary arteries with no focal culprit and preserved left ventricular function. Post the angiogram, he had transient hypotension and required admission to the intensive care unit for inotropic support.

**Figure 2:**
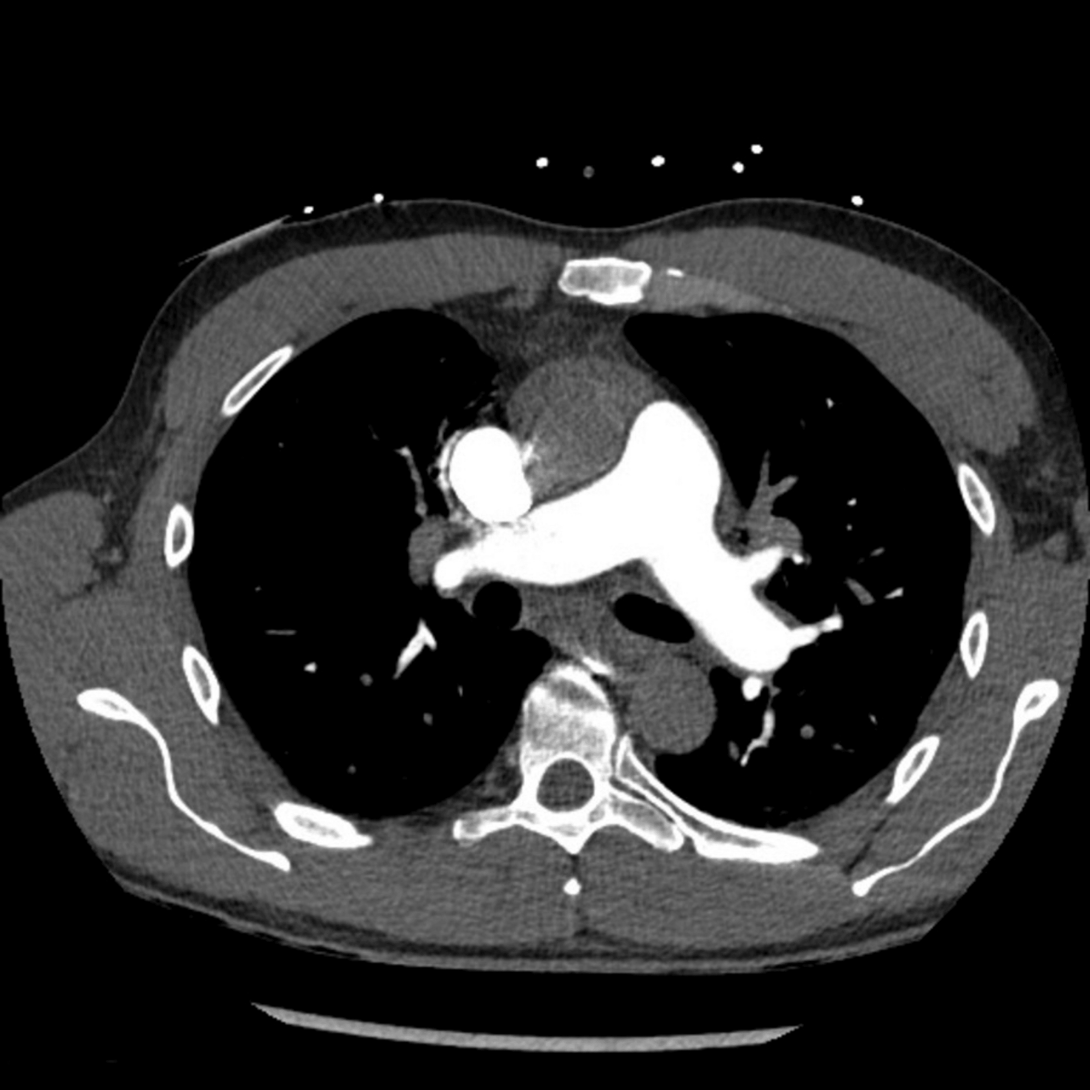
Computed tomographic pulmonary angiography (CTPA) showed no pulmonary embolism.

During the admission, he had thorough investigations, but no clear cause was found for his transient ST elevation and troponin rise. Transthoracic echocardiogram demonstrated normal left ventricular size and systolic function, with a left ventricular ejection fraction of 56%. Computed tomographic pulmonary angiography showed no pulmonary embolism (Figure 2).

**Figure 3:**
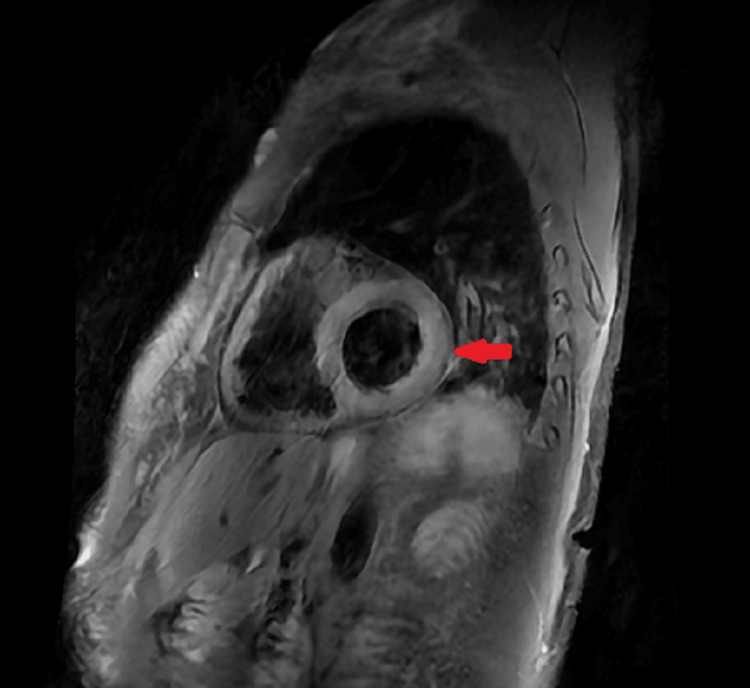
Cardiac MRI T2-weighted black blood image showed no increased signal in the cardiac wall (red arrow), suggesting no myocardial oedema.

The cardiac MRI was normal, with no oedema and no late gadolinium enhancement or fibrosis (Figure 3).

He had abnormal liver function tests, likely related to alcohol (Table 2).

**Table 2:** Liver function test summary. Liver function was significantly impaired on day one, and then improved on discharge. On presentation, day one, and day two, the AST/ALT ratio was greater than 2, along with elevated GGT, indicating alcohol-associated liver disease. ALT: alanine aminotransferase; AST: aspartate aminotransferase; ALP: alkaline phosphatase; GGT: gamma-glutamyl transferase.

Test time	On presentation	Day 1	Day 2	On discharge	Reference range	
Bilirubin (µmol/L)	7	33	23	13	0–20	
ALT (U/L)	131	3421	2356	1125	0–55	
AST (U/L)	421	7185	6361	449	0–40	
ALP (U/L)	114	157	107	101	30–110	
GGT (U/L)	372	527	355	339	0–50	
Total protein (g/L)	56	56	45	61	60–80	
Albumin (g/L)	32	32	26	33	36–49	

**Figure 4:**
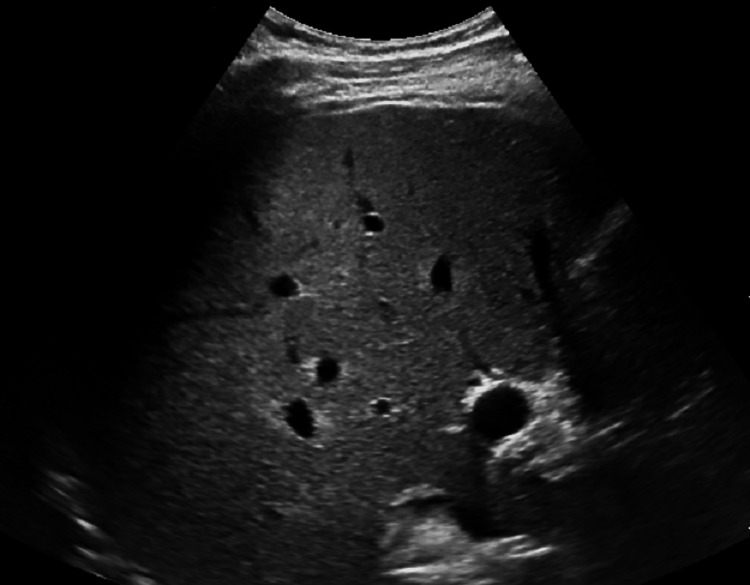
Liver ultrasound showed hepatic steatosis but no cirrhosis.

Liver ultrasound showed hepatic steatosis, but no features of established cirrhosis or portal hypertension (Figure 4).

**Figure 5:**
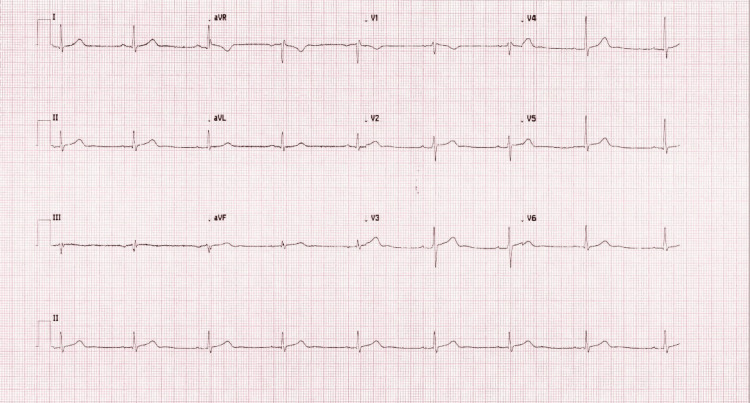
Electrocardiogram (ECG) showed resolution of ST elevation and wide QRS complexes.

Entrectinib was withheld on presentation. He did not experience any further chest pain, and the ST elevation resolved after withholding entrectinib for two days (Figure 5).

He was discharged home after a one-week hospital stay. Entrectinib was withheld for a total of three weeks. He was then reviewed in the outpatient clinic and recommenced entrectinib at a reduced dose of 400 mg daily. He wanted to quit alcohol completely and was referred to an alcohol counselling service.

## Discussion

The exact mechanism for entrectinib-related ST elevation is not yet fully understood. However, it may be due to dysfunction of iron channels and the blockage of depolarization in sodium and calcium channels. One study investigated the impact of entrectinib on sodium currents in cardiomyocytes and found that 48 hours of entrectinib treatment decreased peak sodium current and shifted the voltage dependence of inactivation [3]. Furthermore, the alterations in sodium current do not result from direct channel inhibition but rather from other pathways yet to be defined [3].

There are six published case reports of entrectinib-associated ECG changes (Table 3).

**Table 3:** Summary of case reports. ECG: electrocardiogram; LVEF: left ventricular ejection fraction; MRI: magnetic resonance imaging; CTPA: computed tomography pulmonary angiogram; PE: pulmonary embolism.

Reference number	Age (years), gender	Duration of entrectinib treatment	ECG changes	Troponin rise	Coronary angiogram	LVEF	Cardiac MRI	Endomyocardial biopsy	Symptoms resolved after cessation of entrectinib
This case	56, Male	5 years	ST elevation	Yes	Normal	Normal	Normal	Not done	Yes
[4]	59, Male	3 days	ST elevation, Brugada	Yes	Normal	Normal	Normal	Not mentioned	Yes
[5]	81, Male	3 days	ST elevation, Brugada, ventricular tachycardia	Yes	Normal	Normal	Not mentioned	Not mentioned	Yes
[6]	45, Female	4 days	ST elevation	Yes	Normal	Normal (right heart failure, CTPA no PE)	Normal	Not mentioned	Yes
[7]	27, Male	2 weeks	Ventricular tachycardia	Yes	Not mentioned	Reduced	Not mentioned	Lymphohistiocytic myocarditis	Yes (treated with steroids for myocarditis)
[8]	65, Male	12 days	ST elevation, ventricular tachycardia	Yes	Normal	Normal	Normal	No inflammation or cardiomyocyte injury	Yes
[9]	72, Male	20 days	T wave inversion, left bundle branch block	Not mentioned	Not mentioned	Reduced	Oedema, myocarditis, heart failure, no myocardial infarction	Not mentioned	Yes (treated with steroids for myocarditis)

The patients' ages range from 27 to 81 years, with five males and one female. In all cases, ECG changes occurred shortly after starting entrectinib treatment, within three to 20 days. Our case reported a 56-year-old male patient who developed ST elevation after five years of continuous entrectinib treatment. This late onset of ECG changes after such a prolonged treatment duration has not been reported previously, and the underlying mechanism remains unclear. Further study is needed to determine if the event was caused by changes in the patient's overall condition over time or by cumulative drug effects. The patient had no recent changes to his medications prior to this event.

It is uncertain whether heavy alcohol intake contributed to this event, as alcohol binge drinking can cause coronary artery spasm and ST elevation [10]. Notably, the patient had intermittent binge drinking while on entrectinib in the past without any cardiac complications. Although there are no well-documented direct interactions between entrectinib and alcohol, caution is advised when combining alcohol with entrectinib; both substances can cause hepatotoxicity and potentially exacerbate cardiac arrhythmias.

In all six published cases, entrectinib was discontinued permanently. In our case, we recommenced at a reduced dose of 400 mg daily after withholding it for three weeks. We understand the potential risk of rechallenging entrectinib, even at a reduced dose; however, the patient had a complete radiological response to entrectinib previously, and alternative treatment options were limited. Also, alcohol might be a potential contributing factor to this event. Therefore, we decided to resume entrectinib with regular follow-up in the condition that the patient had agreed to complete alcohol abstinence.

## Conclusions

This case describes a 56-year-old male patient who developed new-onset ST elevation after taking entrectinib for five years for ROS1 fusion-positive NSCLC in the setting of alcohol intoxication. The ST elevation resolved after withholding entrectinib for two days, and no other cause was identified, suggesting that entrectinib may be the potential cause. It is unclear why this event occurred after five years of treatment; maybe alcohol consumption was a contributing factor. However, we cannot confirm this based on a case report. Further research is needed to investigate the long-term cardiac toxicity of entrectinib and the potential interactions between entrectinib and alcohol.
